# English-learning stress and performance in Chinese college students: A serial mediation model of academic anxiety and academic burnout and the protective effect of grit

**DOI:** 10.3389/fpsyg.2022.1032675

**Published:** 2022-11-30

**Authors:** Liling Xu, Zhenhai Wang, Zhiyuan Tao, Chengfu Yu

**Affiliations:** ^1^School of Foreign Studies, South China Normal University, Guangzhou, China; ^2^Center for Studies of Psychological Application, School of Psychology, South China Normal University, Guangzhou, China; ^3^Department of Psychology and Research Center of Adolescent Psychology and Behavior, School of Education, Guangzhou University, Guangzhou, China

**Keywords:** English-learning stress, academic anxiety, academic burnout, English academic performance, grit, Chinese university students

## Abstract

**Introduction:**

Having to adapt to a new environment with various other challenges while completing their studies, Chinese college students experience intense stress related to the study of the English language. However, there has been little research on the serial mediating mechanism of English-learning stress on English academic performance.

**Methods:**

Present study recruited 1130 undergraduate students to finish self-report online questionnaire to collect the information about their English-learning stress, academic anxiety and burnout, English academic performance and grit. We constructed a moderated serial mediation model to test the effect of academic anxiety and academic burnout and explored whether grit can restrict the decrease in academic performance caused by English-learning stress.

**Results:**

Results show that: (1) both academic anxiety and academic burnout mediate the relationship between English-learning stress and performance; (2) academic anxiety and academic burnout show a significant serial mediating role between academic pressure and English academic performance; and (3) grit significantly moderates the relationship between academic burnout and English academic performance.

**Discussion:**

These results lead us to believe that cultivating the grit of Chinese college students may be an effective way to improve the academic performance of those experiencing high English-learning stress.

## Introduction

Although stress is common in all areas of people’s daily lives, its level among college students seems to be particularly high as they face many different challenges (Pedersen and Jodin, 2016), including constantly exposure to unfamiliar professional courses, adapting to school environment, getting along with classmates, and extracurricular activities and works (Larson, 2006). Previous studies have pointed out that learning stress is ever present in university students (Trigueros et al., 2020a).

Among their many stressors, learning-related ones seem to be the most common and critical for college students (Hurst et al., 2013). Learning stress refers to the psychological strain or distress resulting from exposure to the demands of learning situations (Yang and Chen, 2016). Persistent learning stress can lead to the depletion of an individual’s resources and further produce a series of adaptation problems (Bedewy and Gabriel, 2015). Previous studies have shown that learning stress is positively correlated with poor academic performance (Samaha and Hawi, 2016), poor adjustment to university life (Belay Ababu et al., 2018), increased dissatisfaction with life (Karaman and Watson, 2017), and higher risk of depression (Barker et al., 2018; Asif et al., 2020).

The study of the English language is compulsory for Chinese college students, who must all engage in English courses and prepare for a series of English proficiency tests such as the national College English Test (CET-4, CTE-6), International English Language Testing System (IELTS), and Test of English as a Foreign Language (TOEFL), irrespective of their major. Therefore, English learning stress is an important stress source with a wide range of influence. Academic performance in the English language is not only important for their graduation qualifications, but also for their further education and search for employment (Wang et al., 2021; Wu and Tarc, 2021). Thus, the study of English learning stress is a significant topic to which every college student and educator pays close attention, and that also brings considerable negative impact, sometimes to the extent of hindering learners’ improvement in this area (Huan et al., 2008).

Although many studies have confirmed the negative relationship between English learning stress and academic performance (Samaha and Hawi, 2016; Wunsch et al., 2021), the reason behind this is still need further exploration. Given the importance of English learning and the prevalence of related stress, it is necessary to further discuss the mechanism of influence between them.

Academic anxiety refers to uneasy or unpleasant psychological reflection produced by the learning process, often caused by internal conflict, and is a state of tension specific to students (Pekrun, 2005). Many studies have shown that learning stress is one of the most important reasons for academic anxiety among college students (Leung et al., 2010; Tholen et al., 2022; Zhang et al., 2022). For example, Zhang et al. (2022) found that English-learning stress was significantly positively associated with anxiety symptoms in a sample of 1,309 Chinese college students.

Moreover, previous studies have found that emotional anxiety has a high likelihood of affecting a wide range of cognitive processes such as attention, memory storage and retrieval, and problem-solving (Clore and Huntsinger, 2009; Moran, 2016). Consequently, learning anxiety in students may increase their difficulties in the learning process, such as in paying attention, and further lead to a decline in academic performance (Pekrun et al., 2017). In line with this view, many studies have found that learning anxiety is negatively associated with academic performance among students (Chang and Beilock, 2016; Tomasetto et al., 2021; D'Agostino et al., 2022). Therefore, we suggest that learning anxiety is an important mediator between English-learning stress and performance.

Academic burnout refers to a negative attitude and behavior toward studying, experienced as feeling tired of learning, due to excess pressure or a lack of interest (Salmela-Aro and Upadyaya, 2014). According to the demands-resources model, the emergence of academic burnout is caused by an overload of demands that individuals are unable to manage (Demerouti et al., 2001; Salmela-Aro and Upadyaya, 2014). With learning as their primary task, students are usually under the pressure of high academic expectations, created either by themselves or by the outside world. This leads to academic stress that may overwhelm students’ spirit and even lead to burnout (Jiang et al., 2021).

Learning content perceived as highly stressful may make learners tired (Noh et al., 2019). Especially when studying English, learners may experience more pressure due to psychological, social, and culture factors. Consequently, academic burnout is more common among English learners than other students (Khattak et al., 2011). When it becomes chronic, such stress can cause many psychological and physiological problems, including emotional fatigue, irritability or headaches, as well as mental impairment such as memory and attention difficulties (Li, 2019). Furthermore, under high pressure, some students may develop negative perceptions about the course, homework, and teachers (Romano et al., 2020). They may start to think that the learning content is useless and begin to feel alienated in the classroom, stop doing their homework, become hostile to the teachers, and finally tire of their studies (Tennant et al., 2015).

Extant studies have reported that students’ burnout may result in a lack of interest in class activities, feelings of depression, multiple absences, chronic tardiness, and irresponsible behaviors (Kim et al., 2015; Aghajani Liasi et al., 2021; Hwang and Kim, 2022). These difficulties are likely to lead to poor overall academic performance. In addition, individuals with higher levels of academic burnout report lower resilience, and may thus have more difficulty “bouncing back” from negative experiences of academic failure. Being stuck in the aftereffect of failure for long periods may reduce their level of effort going forward, leading to poorer academic performance (Li, 2019; Wang et al., 2021). From the above discussion, it is easy to see that academic burnout is likely to be a mediating variable between English-learning stress and academic performance.

On the premise that the above two mediating mechanisms are established, further exploring the serial mediating mechanism of academic anxiety and academic burnout between academic pressure and achievement can provide a more complete variable relationship between academic pressure and achievement and help explore new effective protective mechanisms. Existing studies hold that moderate levels of anxiety may be beneficial as the driving force for effective learning of new materials, while negative effects are associated with excessive academic anxiety (Romano et al., 2020).

Indeed, previous research has highlighted that students with high levels of anxiety are more likely to view upcoming assignments and exams as potential threats, and report poorer emotional intelligence and less use of emotion regulation strategies (Dobos et al., 2021). Therefore, students with high academic stress find it more difficult to deal with the negative emotions brought on by learning and suffer burnout (Fiorilli et al., 2020). Furthermore, students with severe anxiety tend to avoid social interactions and show lower social and emotional competences, preferring avoidance over support-seeking strategies (Li et al., 2020). These students are less receptive to teachers’ emotional support and gradually rebel against them and become bored with learning (Romano et al., 2020). The foregoing content has already explained the mediating effect of academic burnout and anxiety. Thus, we have reason to assume that there is a positive regression relationship between academic anxiety and burnout, and that they play a serial mediating role between English-learning stress and performance.

Although English-learning stress has a negative impact on performance in most cases, some people with positive individual traits may be able to maintain good performance in stressful situations (Zhou, 2015; Cao and Meng, 2020). Grit is a positive personality trait composed of the “passion and persistence for long-term goals” and the “long-term consistency of interests” (Duckworth et al., 2007; Schimschal et al., 2021). Duckworth et al. (2007) pointed out that grit is developed over a lifetime and motivates individuals in their learning process to overcome stress and failure in pursuit of success. Grit, especially under its aspect of persistence, has been widely found to be associated with students’ higher academic performance (Crede et al., 2017; Liu and Wang, 2021; Lam and Zhou, 2022). Students with high level of grit often report more learning engagement and are more likely to achieve better academic standards (Wolters and Hussain, 2015; Aparicio et al., 2017). In the field of English learning, grit is considered to play a key role (Liu and Wang, 2021; Pawlak et al., 2022; Teimouri et al., 2022). Wei et al. (2019) found that grit positively affected middle-school students’ English academic performances by promoting their learning enjoyment.

In addition to its direct relationship with academic performance, grit is also considered to alleviate the negative impact of an adverse environment on academic performance (Yang and Kyung, 2019; Zhang et al., 2021; Sulla et al., 2022). Duckworth and Duckworth (2016) pointed out that people with a high level of grit are more optimistic and less discouraged when faced with adversity and therefore more likely to record achievements under adverse conditions (e.g., high academic burnout). Similarly, Sulla et al. (2022) found that grit could moderate the relationship between psychological distress and academic performance among university students during the COVID-19 pandemic. Therefore, it can be expected that when English-learning stress leads to academic anxiety and further to academic burnout, students with more grit are more likely to persist in learning and overcome burnout to produce good English academic performances.

**Figure 1:**
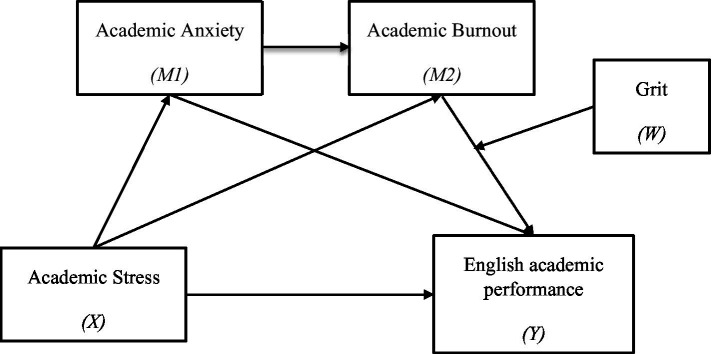
The proposed moderated mediation model.

Overall, the purpose of this study was to examine the effect of English-learning stress on academic performance among college students and further explore the serial mediating roles of learning anxiety and academic burnout. Figure 1 illustrates the proposed research model. Our hypotheses were as follows:

*H1*: Academic anxiety can significantly mediate the relationship betwdeen English-learning stress and English academic performance.

*H2*: Academic burnout can significantly mediate the relationship between English-learning stress and English academic performance.

*H3*: Academic anxiety and academic burnout can serially mediate the relationship between English-learning stress and English academic performance.

*H4*: Grit can moderate the indirect links between academic burnout and English academic performance.

## Materials and methods

### Participants

Participants for the study were recruited using an anonymous online survey administered to college students across a first-tier city in southern China. After eliminating invalid questionnaires (e.g., completed in less than 180 s), a total of 1,130 participants remained including 406 men (35.9%) and 724 women (64.1%), aged between 17 and 25 years (*M* = 20.84, SD = 1.57).

### Measures

#### English-learning stress

English-learning stress was assessed through four items extracted and adapted from previous questionnaires on academic stress (Tholen et al., 2022; Zhang et al., 2022). Participants were required to indicate the extent to which they had felt stressed about several aspects of English learning over the past year, such as completing English courses and assignments and passing the course test or level test (e.g., I feel stressed by the English course examination). All items were rated on a 5-point scale ranging from 1 (never) to 5 (always). Scores were averaged to create a composite score, with higher scores indicating higher English learning stress. In this study, the measure demonstrated excellent reliability (Cronbach’s *α* = 0.907).

#### Academic anxiety

Academic anxiety was assessed using a 7-item questionnaire adapted from the Chinese version of the Foreign Language Classroom Anxiety Scale (Jin and Dewaele, 2018). Participants were asked to evaluate whether they had experienced any anxiety symptoms when learning English, such as worry (e.g., I feel nervous because I cannot understand English), nervousness, and fear of being asked questions in class. All items were rated on a 5-point scale ranging from 1 (strongly disagree) to 5 (strongly agree). The scores were averaged to create a composite score, with higher ones indicating a higher degree of English-learning stress. In this study, the measure demonstrated excellent reliability (Cronbach’s *α* = 0.913).

#### Academic burnout

Learning burnout was assessed using a 13-item questionnaire adapted from the Learning Burnout Questionnaire for Chinese college students (Lian et al., 2005). It consisted of three dimensions: personal accomplishment (e.g., “It is easy for me to master professional knowledge”), depression (e.g., “I feel exhausted after studying all day”), and improper behavior (e.g., “I seldom study after class”). All items were rated on a 5-point scale ranging from 1 (strongly disagree) to 5 (strongly agree). The scores were averaged to create a composite score and negatively worded items were recoded, with higher scores indicating a higher degree of English-learning burnout. In this study, the measure demonstrated good reliability (Cronbach’s *α* = 0.761).

#### Grit

Grit was assessed *via* the 8-item Short Grit Scale, Grit-s (Duckworth and Quinn, 2009), which comprises two dimensions, namely, “perseverance of effort” (e.g., “I am diligent”) and “consistency of interests” (e.g., “New ideas and projects sometimes distract me from previous ones,” reverse code). All items were rated on a 5-point scale ranging from 1 (strongly disagree) to 5 (strongly agree). The scores were averaged to create a composite score, and negatively worded items were recoded to make sure that higher scores indicated higher level of grit. Grit-S has been proven reliable by numerous studies over the past decade in samples from different cultures and age groups (Mullen and Crowe, 2018; He et al., 2021; Schmidt et al., 2021). In this study, the measure demonstrated good reliability (Cronbach’s *α* = 0.810).

#### English academic performance

Participants were asked to provide their average examination scores for English courses. The scores were recorded based on the interval: 1 = <60, 2 = 60–69, 3 = 70–79, 4 = 80–84, 5 = 85–89, 6 = 90–94, 7 = 95–100.

### Covariates

We collected a set of socio-demographics as covariates, including gender and age. Additionally, participants were asked to report their performance in major courses and their general stress level as covariates.

### Consent and ethical considerations

The study was approved by the ethics committee of the author’s affiliated university. Participants were asked to read and confirm their agreement on an informed consent statement. Participating adolescents took about 15 min to complete the entire series of self-report questionnaires online. They were informed that their participation was voluntary and they could withdraw at any time.

### Statistical analyses

Data analysis was conducted in four steps using IBM SPSS Statistics for Windows, version 26.0. First, Harman’s single-factor test was used to investigate the possibility of common method bias and found that 13.55% of the variance could be explained by the first principal factor, which was far less than the threshold of 40% (Lee et al., 2011), indicating no common method variance. Second, descriptive statistics and Pearson’s correlation analysis were performed. Third, SPSS macro PROCESS 3.3, Model 8 (Hayes, 2013), with a 95% bias-corrected confidence interval (CI) based on 5,000 bootstrap samples was used to examine the mediator and moderator in the models. In recent psychological research, PROCESS has been adopted as a universal and effective model analysis method. Hayes states that there is little difference in the results obtained through PROCESS and SEM, and rarely will the substantive conclusions a researcher arrives at be influenced by the decision to use one over the other. Finally, when the moderating effect was significant, we drew a simple slope diagram based on the values obtained by adding or subtracting one standard deviation from the moderating variable.

## Results

Descriptive statistics and correlations for all variables are presented in Table 1. As expected, English-learning stress is positively associated with academic anxiety, academic burnout, and poor English academic performance. In addition, academic anxiety and burnout were negatively associated with English academic performance. Grit also negatively correlates with English-learning stress, anxiety, and burnout while positively associating with English academic performance. These results support our subsequent model construction.

**Table 1:** Means, standard deviations, and correlation coefficients for all variables.

Variables	1	2	3	4	5	6	7
1. Gender	–						
2. Age	−0.00	–					
3. Stress	0.06*	−0.02	–				
4. Anxiety	0.02	−0.02	0.59***	–			
5. Burnout	0.01	−0.07*	0.38***	0.53***	–		
6. English	−0.12***	−0.03	−0.29***	−0.38***	−0.26***	–	
7. Grit	−0.03	0.07*	−0.08***	−0.16***	−0.33***	0.20***	–
*Mean*	0.36	20.27	3.00	2.90	2.95	3.30	3.07
*SD*	0.48	1.48	1.03	0.92	0.44	1.50	0.77

N = 1,130, gender was dummy coded such that 1 = male, 0 = female. Stress, English-learning stress; Anxiety, academic stress; Burnout, academic burnout; English, English academic performance; *p < 0.05, ***p < 0.001.

### Moderated serial mediation analyses

**Figure 2:**
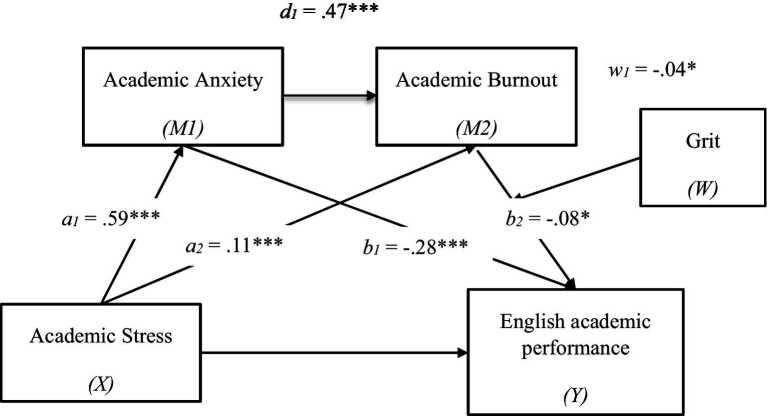
The result of moderated serial mediation model, *p < 0.05, ***p < 0.001. The values shown are standardize coefficients.

Results of the moderated serial mediation analyses are presented in Figure 2. First, the results of Model 1 (Academic anxiety as outcome variable, *R^2^* = 0.36, *F* = 205.11, *p* < 0.001), Model 2 (Academic burnout as outcome variable, *R^2^* = 0.31, *F* = 121.01, *p* < 0.001), and Model 3 (English academic performance as outcome variable, *R^2^* = 0.18, *F* = 35.15, *p* < 0.001) were all significant. We found a negative direct effect of English-learning stress on English academic performance (total effect: *β* = −0.29, *p* < 0.001). When the mediators were included in the analysis, this coefficient decreased but was still statistically significant (direct effect, *β* = −0.09, *p* < 0.01).

Confirming Hypotheses 1 and 2, English-learning stress is also found to be a positive predictor of academic anxiety (*β* = 0.59, *p* < 0.001) and academic burnout (*β* = 0.11, *p* < 0.001). Additionally, English-learning stress (*β* = −0.28, *p* < 0.001) and academic burnout (*β* = −0.08, *p* < 0.05) were, respectively, positively related to English academic performance, and the indirect effect of the pathway, “English-learning stress → academic anxiety → English academic performance” (*β* = −0.16, *SE* = 0.03, 95% CI = [−0.22, −0.11]), is significant and can explain 57.14% of the variance in the relationship of English-learning stress and English academic performance. The pathway, “English-learning stress → academic burnout → English academic performance” (*β* = −0.01, *SE* = 0.00, 95% CI = [−0.02, −0.00]) is also statistically significant and can explain 3.57% of the variance.

**Figure 3:**
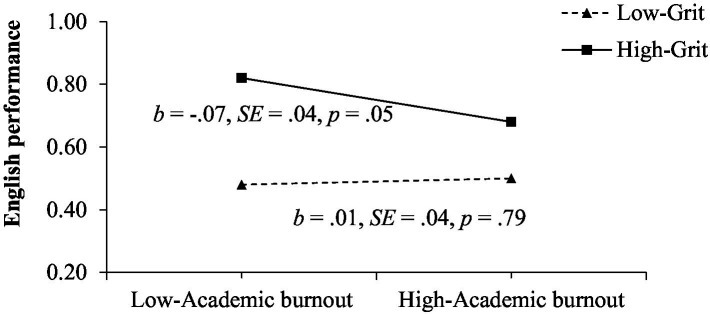
English academic performance as a function of academic burnout and grit.

Moreover, our results also support Hypotheses 3 and 4: academic anxiety is a positive predictor of academic burnout (*β* = 0.47, *p* < 0.001), and the serial mediation pathway, “English-learning stress → academic anxiety → academic burnout → English academic performance” (*β* = −0.02, *SE* = 0.01, 95%CI = [−0.04, −0.00]) is marginally significant and can explain 7.14% of the variance. The effect of the interaction between academic burnout and grit on English academic performance is significant (*β* = −0.04, *p* = 0.05). For clarity, simple slope tests were used to further investigate the effect of grit. These results, presented in Figure 3, indicate that the relationship between academic burnout and English academic performance is significant among highly gritty college students (*β* = −0.07, *p* = 0.05) but insignificant among students with low grit.

## Discussion

This study examined the potential mediating role of learning anxiety and learning burnout in the relationship between English-learning stress and academic performance as well as the moderating role of grit.

Firstly, our results support the inference that English-learning stress negatively affects English academic performance. A possible explanation is that, compared with senior high school students, college students face greater pressure, such as adapting to a new environment and interpersonal problems (Pedersen and Jodin, 2016). Thus, the increased pressure from studying English may overwhelm them and lead to poor English academic performance. More importantly, this study identified three different mediating mechanisms between English-learning stress and English academic performance, which may be helpful for educators to understand the reason for the decline in students’ English academic performance and then formulate targeted interventions for improvement.

Consistent with Hypothesis 1, academic anxiety can partly mediate the relationship between English-learning stress and English academic performance, which reveals that high English-learning stress may lead to more academic anxiety and impact English academic performance. Anxiety is a normal psychological response to pressure (Dyson and Renk, 2006). Students who are chronically experiencing high learning stress are more likely to feel diffident about their academic abilities and develop emotional anxiety about academic activities and examinations. Our study confirms the conclusion of a previous work, that learning stress can lead to academic anxiety and poor academic performance (Iqbal et al., 2015).

Consistent with Hypothesis 2, English-learning stress is positively correlated with academic burnout, which is consistent with previous research results (Kilic et al., 2021; Yusoff et al., 2021). We also believe that moderate English-learning stress can effectively induce college students’ curiosity about English learning, cultivate their active learning motivation, mobilize their learning potential, and therefore, smoothly improve their learning efficiency and ultimately improve their English learning performance (de la Fuente et al., 2020). However, once the academic stress exceeds the load-bearing potential of the students, they will not be able to effectively self-regulate, making them susceptible to high academic burnout. In an environment of high learning pressure, students may form negative cognition about their schoolwork and teachers, question the role and significance of learning English, and lose interest in learning (Romano et al., 2020). Moreover, college students’ sense of self-efficacy may be damaged under high academic stress and eventually cause them to report higher academic burnout rates. In such a case, individuals may think that they are not able to complete English-learning tasks and may eventually begin to have multiple psychological and behavioral problems, such as evasion and boredom with daily learning tasks and examinations, all of which have a negative impact on their English academic performance (Aghajani Liasi et al., 2021). Meanwhile, academic burnout is significantly positively related to students’ physical and emotional fatigue (Li, 2019). This may affect the individuals’ daily lives, besides their attention to learning tasks and examinations, and lead to poor English academic performance.

The serial mediation mechanism is also in line with our Hypothesis 3: we can see that English-learning stress is significantly positively related to poor English academic performance *via* academic anxiety and academic burnout. Anxiety is often accompanied by stress, and the degree of anxiety rises with the increase in stress levels. When students bear too much academic pressure, the resulting anxiety will reach a high level and damage students’ enthusiasm for learning, thus positively relating to academic burnout (Trigueros et al., 2020b). Individuals with high academic anxiety may be more likely to regard future English academic tasks and exams as potential threats and, therefore, to have negative cognition about their studies, which is ultimately positively related to academic burnout (Zhou, 2021). Especially in the context of the normalization of COVID-19 pneumonia monitoring in China, the academic stress is constantly increasing among college students (Yang and Yang, 2022). With the growing uncertainty of what form the English classroom may take, and the reformed teaching methods during COVID-19 pandemic, students are more likely to face greater academic stress. It is thus even more necessary for our serial mediation to explore the possible mechanism of English-learning stress affecting English academic performance. Our findings can help identify ways to reduce academic anxiety and burnout and to avoid the decline in English academic performance.

The last conclusion of this study and the most important one is that grit could moderate the mediating pathways from English-learning stress to English academic performance. Specifically, students with a high level of grit show better English academic performance than those with low levels; however, this difference is larger when they report a low level of academic burnout. This result, which is not completely consistent with our expectations, may be reasonably explained by the fact that one important reason why grit could affect academic performance is that it promotes persistence in pursuing academic goals (Lam and Zhou, 2022). However, students with high academic burnout are more likely to give up on their academic goals in English learning, thus weakening the protective effect grit has on English academic performance. Moreover, though college students have to pass a series of examinations to obtain graduation qualifications, learning is not the only important activity during college life. Students with high academic burnout may devote their energy to other activities, such as engaging in practical programs, establishing an interpersonal relationship, or participating in sports activities. At this time, it is difficult to reflect on the protective role of grit on English academic performance. However, regardless of the level of academic burnout, students with higher grit achieve better English academic performance than those with low grit, which emphasizes the importance of developing this personal quality in college students.

### Limitations

The first limitation of this study is that we only considered grit as a single individual protection factor. It is reasonable to use a variety of research methods to collect more detailed data about other positive psychological characteristics or states (e.g., resilience, motivation) as buffers, to further investigate the protective factors for perfecting the protection mechanism against college students’ decline in English academic performance. Besides, previous research shows that types of learning motivation (especially controlled motivation) are closely related to academic anxiety, academic burnout, and English academic performance, suggesting that including learning motivation in the theoretical model could yield better results. Secondly, the data were collected in a low-risk sample; further, the serial mediating mechanism established in college students may not be extendable to children and early adolescents. Last but not least, using a longitudinal and cross-lagged model with multiple informants (e.g., teachers, parents, and peers) to confirm the relationships between these variables would have been more effective than cross-sectional research. Although cross-sectional data is our disadvantage, our new exploration on the mediating mechanism and protective factors is still of value.

### Implications

Despite its limitations, our study has several meaningful implications for university English education. First, our findings suggest that English-learning stress has a negative association with English academic performance. Therefore, English educators could guide their college students to set appropriate learning goals to avoid excessive learning stress in the short term. We also found three different mediation mechanisms between English-learning stress and English academic performance, which suggest that alleviating anxiety aroused by learning stress and thus avoiding burnout is a key process to avoiding the decline in English academic performance. An appropriate approach is to develop students’ coping strategies and regulate their ability to manage stressful situations, helping them reduce the generation of emotional anxiety when faced with learning stress. In addition, for students who are already in a state of academic anxiety, educators could reduce the possibility of burnout by cultivating their interest in learning or stimulating their growth initiative. Lastly, we also highlight the key role of grit in English learning. As a malleable trait (Duckworth et al., 2007), grit can be developed through school education to help students better cope with stress, anxiety, burnout, and other unfavorable factors of learning, and acquire good academic performance.

## Data availability statement

The raw data supporting the conclusions of this article will be made available by the authors, without undue reservation.

## Author contributions

LX and ZW wrote the first draft of the manuscript and commented on previous versions of the manuscript. CY and ZT performed the material preparation, data collection, and analysis. All authors contributed to the conception and design, read, and approved the final manuscript.

## Conflict of interest

The authors declare that the research was conducted in the absence of any commercial or financial relationships that could be construed as a potential conflict of interest.

## Publisher’s note

All claims expressed in this article are solely those of the authors and do not necessarily represent those of their affiliated organizations, or those of the publisher, the editors and the reviewers. Any product that may be evaluated in this article, or claim that may be made by its manufacturer, is not guaranteed or endorsed by the publisher.
